# Co-occurring self-efficacy, anxiety, and depression in caregivers of patients with heart failure: A Group-Based Dual-Trajectory Modeling Approach

**DOI:** 10.1186/s12912-025-02995-0

**Published:** 2025-03-31

**Authors:** Qingyun Lv, Hairong Chang, Yaqi Wang, Xueying Xu, Jingwen Liu, Yuan He, Li Fu, Mei Lin, Xinxing Gao, Xiaoying Zang, Xiaonan Zhang

**Affiliations:** 1 School of Nursing, Tianjin Medical University, No. 22 Qixiangtai Road, Heping District, Tianjin, 300070 China; 2 Department of Nursing, The Second Hospital of Tianjin Medical University, Tianjin, China; 3 Department of Nursing, Tianjin Medical University General Hospital, Tianjin, China; 4 Department of Cardiology, The Second Hospital of Tianjin Medical University, Tianjin, China

**Keywords:** Heart failure, Caregivers, Self-efficacy, Depression, Anxiety

## Abstract

**Background:**

Self-efficacy and mental health present mutually influencing relationships in caregivers of patients with heart failure (HF). There is currently a lack of understanding the synergistic developmental mechanisms of self-efficacy, anxiety, and depression in caregivers. The purpose of the study was to examine the individual and dual trajectories of caregiver self-efficacy, anxiety, and depression during the first three months after the discharge of patients with HF.

**Methods:**

A prospective cohort study was conducted from June 2022 to May 2024, in four tertiary hospitals in Tianjin, China. A total of 299 family caregivers of patients with HF were enrolled in the cohort, and 267 completed follow-ups. Group-based dual trajectory modeling was employed to examine the development of self-efficacy, anxiety, and depression.

**Results:**

The mean (SD) age of caregivers was 58.3 (13.1) years, and 164 (61.4%) were women. Three trajectories of caregiver self-efficacy were identified: low-curve (25.1%), middle-curve (67.8%), and high-stable (7.1%). Regular exercise, work status, and chronic disease were associated with the different caregiver self-efficacy trajectories. A 3-class trajectory solution was chosen for depression and anxiety when analyzed separately. The proportions of ideal combinations of high-stable self-efficacy and initial-to-mild anxiety or depression were extremely small, at 12.60% and 8.00%, respectively. Caregivers had limited and inconsistent abilities to regulate the effects of anxiety and depression on their self-efficacy.

**Conclusion:**

The present study identified three distinct trajectories of self-efficacy, anxiety, and depression among family caregivers of patients with HF. The dual-trajectory models revealed the probability of interrelationships between caregiver self-efficacy trajectories and those of anxiety and depression, suggesting substantial opportunities to enhance caregivers’ self-efficacy and mental well-being of patients with HF.

**Supplementary information:**

The online version contains supplementary material available at 10.1186/s12912-025-02995-0.

## Background

Family caregivers of patients with heart failure (HF) have emerged as valuable partners of the health system, playing a vital role in assisting patients to adopt and maintain self-care behaviors for HF management [1]. Caregiver self-efficacy has been defined as the caregivers’ belief in their ability to assist patients with self-care, which includes helping patients maintain disease stability, promote symptom monitoring and perception, and respond to worsening physical conditions [2]. Caregiver self-efficacy can enhance patient adherence in managing chronic diseases, subsequently leading to positive impacts on patient outcomes [3–5].

The grim reality is that caregiver self-efficacy urgently requires improvement. More than half (54.5%) of HF caregivers have inadequate levels of self-efficacy [6]. Additionally, self-efficacy may vary due to individual characteristics such as physical activity level, work status, and other factors [7, 8]. However, achieving optimal outcomes from interventions aimed at enhancing caregiver self-efficacy remains a significant challenge. Based on the existing evidence, while interventions for caregivers require further unification and standardization, it is clear that caregiver training solely conducted during hospitalization failed to improve caregiver self-efficacy post-discharge [9]. The dynamic nature of caregiver self-efficacy, which can fluctuate based on situational factors, may not have been adequately accounted for in the study design.

It is well-established that the first 3 months following patients’ discharged after an episode of acute HF is referred to as the “vulnerable period” for chronic HF patients [10]. Approximately 30% of patients hospitalized with HF are readmitted in this period, and mortality during this period can approach 10% [11–14]. The “vulnerable period” represents a window of opportunity for intervention. Therefore, it is crucial for clinicians to understand the key trends and temporal milestones in the ‘vulnerable period’ of caregivers across different self-efficacy trajectories before implementing tailored interventions.

Equally important is that caregiver self-efficacy plays a crucial role in the mental health of caregivers after they have taken on their responsibilities. Anxiety and depression are common psychological burdens experienced by caregivers of patients with HF [15]. Caregiver self-efficacy has been well-established as a key predictor and mediator of anxiety or depression [16]. Numerous studies have found that caregivers with high self-efficacy tend to experience lower levels of stress, anxiety, and depression [17, 18]. They are more likely to approach challenges with optimism and resilience, resulting in better mental health outcomes. Additionally, caregiver self-efficacy was found to be a partial mediator between communication with health professionals and psychological distress, as well as a full mediator between trust in health professionals and psychological distress [19]. Researchers also found that the relationship between caregiver burden at an initial time point and depressive symptoms one year later was partially mediated by self-efficacy [20]. Notably, emotional and psychosocial support for caregivers of patients with chronic conditions can somewhat improve their self-efficacy [21]. Thus, self-efficacy and mental health are mutually influencing relationships, with deterioration of either one leading to changes in the other. Additionally, previous research has primarily focused on variable-oriented analyses, overlooking the identification of developmental patterns and characteristics of caregiver self-efficacy latent groups. Overall, based on the current evidence, we still lack a clear understanding of the synergistic developmental mechanisms underlying the simultaneous manifestation of multiple emotional states, including self-efficacy, anxiety, and depression, in caregivers during 3 months after patients’ discharge.

The aim of this study was to examine the individual and dual trajectories of caregiver self-efficacy, anxiety and depression during the first three months after patient discharge. It was hypothesized that (1) distinct trajectories of caregiver self-efficacy, depression and anxiety would be identified; (2) caregiver self-efficacy trajectories would be interrelated to trajectories of depression and anxiety.

## Methods

## Study design and participants

This prospective cohort study was conducted from June 2022 to May 2024, and caregivers of patients with HF were conveniently recruited from the cardiology wards of four tertiary hospitals in Tianjin, China. The inclusion criteria were: the patients they cared for were ≥ 18 years of age and had HF as the primary admission diagnosis. The family caregivers were aged 18 or older, family members or close relatives of the patients, provided the most informal care tasks (e.g., physical, emotional, or financial support) [22]. Additionally, caregivers were required to possess clear awareness, as well as reading and expressive abilities in Mandarin, which is the primary form of Chinese. The exclusion criteria were: the caregivers were paid or participated in any clinical trials within the past three months. Written or verbal informed consent was obtained from the caregivers and the patients they cared for at the time of enrollment. We emphasized in the consent form that participation in this study was voluntary and non-participation would not result in the continuity or quality of HF services. The study protocol was approved by the Ethics Committee of Tianjin Medical University (Grant Number: TMUhMEC2022021). This research study followed the Strengthening the Reporting of Observational Studies in Epidemiology (STROBE) reporting guideline.

The sample size was analyzed using G*power 3.1 software, and the Single-group repeated-measures analysis of variance algorithm was selected [23]. Four measurements were taken. With 95% confidence intervals, a nonsphericity correction (ε) of 0.5, and a power of 0.8, the minimum sample size required for our study was 182. Considering a possible 20% attrition rate, a total of 228 participants were needed.

## Measurement

The sociodemographic variables of both the caregivers and their patients were collected. For caregivers, these variables included age, gender, educational level, relationship to the patients, years of caregiving, work status, residence, lifestyle (alcohol use, tobacco use, regular exercise), and presence of chronic diseases. For patients, the sociodemographic variables included age, gender, educational level, and medical insurance. Health-related factors for the patients included New York Heart Association (NYHA) functional class and comorbidity counts.

Caregiver self-efficacy was assessed using the Caregiver Self-Efficacy in Contributing to Patient Self-Care (CSE-CSC) Scale. This scale was developed by Maddalena De Maria et al. [24]. The Chinese version translated by Lv et al. [25], and take Mandarin in to account. It includes two subscales: self-efficacy in self-care maintenance and monitoring, and self-efficacy in self-care management, which can effectively assess caregiver self-efficacy. The scale uses a 5-point Likert format (1 = “not confident” to 5 = “very confident”), and the total score is standardized on a scale of 0–100. The Cronbach’s alpha of the original version was 0.942 for the whole scale [24]. In the present study, the Cronbach’s alpha value at baseline was 0.932. The Kaiser–Meyer–Olkin (KMO) value was 0.926, and the Bartlett spherical test reached a significance level (χ^2^ = 1996.804, *P* < 0.001). The maximal variance orthogonal rotation method identified two factors with eigenvalue above 1, and the cumulative variance contribution rate was 73.14%. The CSE-CSC showed good reliability and validity in this study.

Caregiver anxiety was assessed using the 7-item General Anxiety Disorder (GAD-7) [26]. This tool identifies individuals with a generalized anxiety disorder and assesses symptom severity. It consists of 7 questions, scored from 0 (not at all) to 3 (nearly every day), with total scores ranging from 0 to 21. According to the GAD-7 manual, scores of 5–9 indicate mild symptoms, 10–14 moderate symptoms, and 15 or higher severe symptoms [26]. The GAD-7 has been translated into Chinese, demonstrating good reliability and validity, evidenced by a Cronbach’s alpha value of 0.888 and a stable one-factor structure [27]. In this study, the baseline Cronbach’s alpha value was 0.892. The KMO score was 0.862, and the Bartlett’s test of sphericity produced an χ^2^ value of 1450.851 (*P* < 0.001). The maximal variance orthogonal rotation method identified one factor with eigenvalue above 1, and the cumulative variance contribution rate was 67.27%. The GAD-7 demonstrated good reliability and validity in our study.

Caregiver depression was measured using the Patient Health Questionnaire-9 (PHQ-9) [28], which assesses depression severity and criteria for major depressive episodes. It consists of 9 items on a 4-point scale. The total scores range from 0 to 27 (5–9: mild; 10–14: moderate; 15–19: moderately severe; ≥ 20: severe) [28]. The PHQ-9 has been translated and validated in Chinese populations [29]. In this study, the Cronbach’s alpha value was 0.880. Besides, the KMO score was 0.878, and the Bartlett’s test of sphericity produced an χ^2^ value of 1471.801 (*P* < 0.001). The maximal variance orthogonal rotation method identified one factor with eigenvalue above 1, and the cumulative variance contribution rate was 71.051%. The PHQ-9 showed good reliability and validity in this study.

## Procedure

Participant assessment occurred at 4 time points: within 24 h before discharge (T0), and 2 weeks (T1), 1 month (T2) and 3 months (T3) after discharge. After obtaining informed consent from the caregivers and their patients, data collection started. Site-specific investigators underwent standardized training and had no personal or professional relationship with the institutions. Paper-based questionnaires were provided to the caregivers, allowing them to complete questionnaires independently during the baseline (T0) survey. If they encountered any writing difficulties, such as upper limb dysfunction, the investigators were available to assist them in filling out their answers on the paper questionnaire. Caregivers were contacted by telephone or email for subsequent assessments (T1–T3). Caregivers who were lost to follow-up or experienced the death of the patient they cared for were defined as dropouts and excluded from the follow-up cohort. If the caregiver lost contact for two consecutive time points, they were defined as lost to follow-up. Data collection occurred through a combination of caregiver interviews by trained investigators and self-reported measures.

## Statistical analysis

In this study, all participants with at least three measurements within the 4 time points were included. Missing data handling was conducted using maximum likelihood estimation, which generates asymptotically unbiased parameter estimates through the assumption that the data are missing at random [30]. Patient and caregiver characteristics were described using mean and standard deviation (*SD*) for normal distributed continuous variables, and frequency and percentage for categorical variables. ANOVA for continuous variables and chi-square tests for categorical variables were used to test for differences in baseline characteristics among the caregiver self-efficacy trajectory groups.

Group-based dual trajectory modeling (GBDTM) was used to identify the multidimensional and dynamic associations in caregiver self-efficacy, depression and anxiety. This study followed the dual trajectory model-fitting procedure recommended by Nagin [30]. First, the univariate trajectories of caregiver self-efficacy, depression and anxiety were formed separately using Group-based trajectory modeling (GBTM). On the time axis T0 was defined as the zero-time point, and months after discharge were used as a timescale for the trajectories. A censored normal distribution was applied, and polynomial models up to the three degrees (intercept, linear, quadratic, and cubic) were tested with 1–5 trajectory groups to explore the best-fitting model. Model choice was based on four indices: (1) Bayesian information criteria (BIC) and the Akaike Information Criteria (AIC) estimate the relative amount of information lost by a given model; a smaller number indicates a better model fit. (2) Entropy value at least 0.7, (3) Odds of correct classification greater than 5.0 for all groups, and (4) Interpretability of the model [31]. Next, the starting parameters from the final univariate models of caregiver self-efficacy, depression and anxiety were used as the initial parameters in the dual trajectory modeling. At this stage, separate dual trajectory models were fitted for caregiver self-efficacy and anxiety, as well as caregiver self-efficacy and depression, to assess whether changes in self-efficacy were concurrently associated with changes in depression or anxiety over time. The three key outputs of the dual model are as follows: (1) the trajectory groups for both measurement series, (2) the probability of membership in each identified trajectory group, and (3) conditional probabilities linking membership across the trajectory groups of the two respective behaviors [32].

In this study, the Cronbach’s alpha coefficient assessed instrument reliability, with values greater than 0.7 indicating good reliability [33]. Exploratory factor analysis evaluated validity, requiring a KMO value over 0.6 and a Bartlett’s test *p*-value below 0.05. Good validity is indicated when the number of extracted factors with eigenvalue above 1 match the scale’s dimensions, along with a cumulative variance contribution rate exceeding 60% [34].

The “traj” plugin in Stata 17.0 was used to conduct GBTM and GBDTM analyses, while IBM SPSS Statistics 24.0 was used for the remaining analyses [32]. All statistical tests were 2-sided, and *P* < 0.05 was considered statistically significant [33].

## Results

**Fig. 1:**
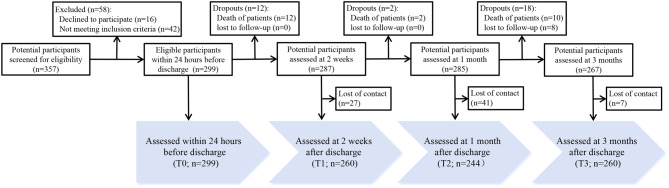
Flow diagram for the data collection procedure

A total of 357 caregivers and their patients were approached and screened for eligibility, and 299 were enrolled. During the 3-month follow-up period, 24 caregivers withdrew due to death and 8 were lost to follow-up (Fig. 1). Finally, 267 caregivers completed 3 or more assessments and were included in subsequent analyses.

The mean (*SD*) age of the 267 enrolled caregivers was 58.3 (13.1) years, 164 (61.4%) were women (Table 1). Forty-five caregivers (15.4%) reported suffering from chronic diseases. The mean (*SD*) age of the patients was 69.5 (12.0) years, and 160 (59.9%) were men. Among the patients, 16.9% had one additional chronic disease, 28.1% had two additional chronic diseases, 27.3% had three additional chronic diseases, and 27.7% had more than four additional chronic diseases. Compared to those included in the current analyses, excluded caregivers were more likely to have lower educational levels and to suffer from chronic diseases (ETable 1).

**Table 1:** Baseline characteristics of the caregivers and their patients within the whole group and across different self-efficacy trajectories

Characteristics	All participants (N = 267)	Caregivers divided into self-efficacy trajectory classes		
		Low-curve group (N = 67)	Middle-curve group (N = 181)	High-stable group (N = 19)
	n (%)/ mean ± SD	n (%)/ mean ± SD	n (%)/ mean ± SD	n (%)/ mean ± SD
Caregivers				
Age (years)	58.3 ± 13.1	59.2 ± 13.1	58.6 ± 13.2	52.3 ± 11.0
Gender				
Men	103 (38.6)	27 (40.3)	70 (38.7)	6 (31.6)
Women	164 (61.4)	40 (59.7)	111 (61.3)	13 (68.4)
Educational level				
Elementary	32 (12.0)	10 (14.9)	22 (12.2)	0 (0.0)
Middle school	104 (39.0)	30 (44.8)	69 (38.1)	5 (26.3)
High school and above	131 (49.1)	27 (40.3)	90 (49.7)	14 (73.7)
Life style^1^				
Alcohol use	46 (17.2)	13 (19.4)	32 (17.7)	1 (5.3)
Tobacco use	65 (24.3)	18 (26.9)	44 (24.3)	3 (15.8)
Regular exercise*	95 (35.6)	16 (23.9)	65 (35.9)	14 (73.7)
Relationship to patients				
Spouse/partner	133 (49.8)	33 (49.3)	92 (50.8)	8 (42.1)
Child	122 (45.7)	28 (41.8)	83 (45.9)	11 (57.9)
Other	12 (4.5)	6 (9.0)	6 (3.3)	0 (0.0)
Times providing care to patient (years)				
<3	34 (12.7)	9 (13.4)	25 (13.8)	0 (0.0)
3–6	95 (35.6)	25 (37.3)	61 (33.7)	9 (47.4)
>6	138 (51.7)	33 (49.3)	95 (52.5)	10 (52.6)
Work status^2^*				
Manual work	95 (35.6)	20 (29.9)	63 (34.8)	12 (63.2)
Brain work	49 (18.4)	15 (22.4)	33 (18.2)	1 (5.3)
Retirement	79 (29.6)	15 (22.4)	61 (33.7)	3 (15.8)
No work	44 (16.5)	17 (25.4)	24 (13.3)	3 (15.8)
Residence				
Urban	220 (82.4)	53 (79.1)	150 (82.9)	17 (89.5)
Town/countryside	47 (17.6)	14 (20.9)	31 (17.1)	2 (10.5)
The presence of chronic diseases*				
No	226 (84.6)	47 (70.1)	163 (90.1)	16 (84.2)
Yes	41 (15.4)	20 (29.9)	18 (9.9)	3 (15.8)
Patients they cared for				
Age (years)	69.5 ± 12.0	68.4 ± 12.3	70.2 ± 11.9	67.0 ± 12.9
Gender				
Men	160 (59.9)	46 (68.7)	102 (56.4)	12 (63.2)
Women	107 (40.1)	21 (31.3)	79 (43.6)	7 (36.8)
Educational level				
Elementary	59 (22.1)	13 (19.4)	43 (23.8)	3 (15.8)
Middle school	128 (47.9)	27 (40.3)	89 (49.2)	12 (63.2)
High school and above	80 (30.0)	27 (40.3)	49 (27.1)	4 (21.1)
Medical insurance				
Having	220 (82.4)	53 (79.1)	151 (83.4)	16 (84.2)
No	47 (17.6)	14 (20.9)	30 (16.6)	3 (15.8)
NYHA class				
I or II	111 (41.6)	24 (35.8)	79 (43.6)	8 (42.1)
III	116 (43.4)	26 (38.8)	82 (45.3)	8 (42.1)
IV	40 (15.0)	17 (25.4)	20 (11.0)	3 (15.8)
Comorbidity counts				
1	45 (16.9)	10 (14.9)	33 (18.2)	2 (10.5)
2	75 (28.1)	14 (20.9)	55 (30.4)	6 (31.6)
3	73 (27.3)	17 (25.4)	53 (29.3)	3 (15.8)
≥4	74 (27.7)	26 (38.8)	40 (22.1)	8 (42.1)

Abbreviations: *NYHA class* New York Heart Association (NYHA) functional class

^1^Alcohol use was defined as drinking more than once a week, with each drinking session involving ≥ 50 ml of alcohol, and a drinking duration of more than 6 months. Tobacco used was defined as smoking ≥ 1 cigarette per day for a duration of more than 6 months. Regular exercise was defined as participating in aerobic exercise ≥ 3 times per week, with each session lasting ≥ 30 min

^2^Manual work included tasks primarily requiring physical exertion, such as agricultural production, craftsmanship, and workers in the service industry. Brain work included professional and technical work such as administrative personnel, salespeople, and office staff

*Represented a significant chi-square or analysis of variance

## Caregiver self-efficacy, anxiety and depression trajectories

**Fig. 2:**
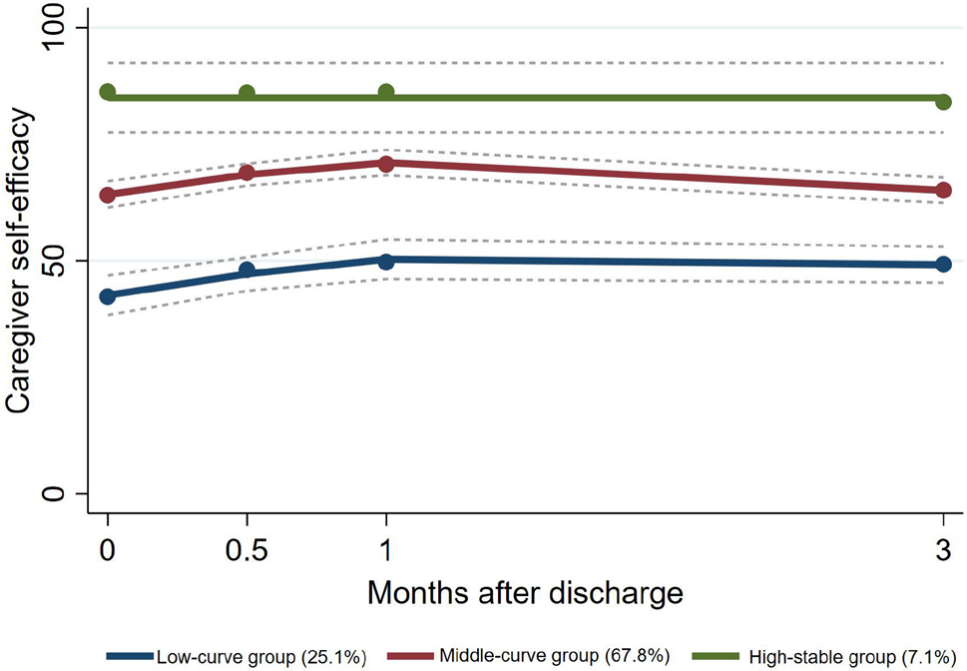
Caregiver self-efficacy Trajectories (N = 267). Solid lines represented the average estimated self-efficacy over time. Dashed lines represented the 95% confidence interval

The fit indices for the 1- to 5- class GBTM models of the caregiver self-efficacy scores are shown in ETable 2. While the information criteria decreased from the 2-class to the 5-class models, and the entropy remained above 0.7, some groups in the 4-class and 5-class models had a ratio less than 5.0%. Based on the model fit indices, the 3-class model was identified as the best-fitting solution. Three distinct caregiver self-efficacy trajectories were identified (Fig. 2). Around one-quarter of caregivers (n = 69) belonged to the low-curve group which had self-efficacy scores below 50 before discharge, followed by an upward trend in the first month after discharge. However, it is noteworthy that there was a slightly decreasing trend from 1 to 3 months after discharge. Approximately two-thirds of caregivers (67.8%, n = 181) were in the middle-curve group, which was characterized by moderate self-efficacy levels and a similar trend to the low-curve trajectory. A total of 7.1% (n = 19) were in the high-stable group, maintaining high self-efficacy levels without changes over time. Univariate analysis showed that compared to the other trajectory groups, the high-stable group had significantly more caregivers who reported regular exercise and manual work (Table 1). Additionally, the low-curve group had a significantly higher chronic diseases incidence rate than that in the other groups.

**Fig. 3:**
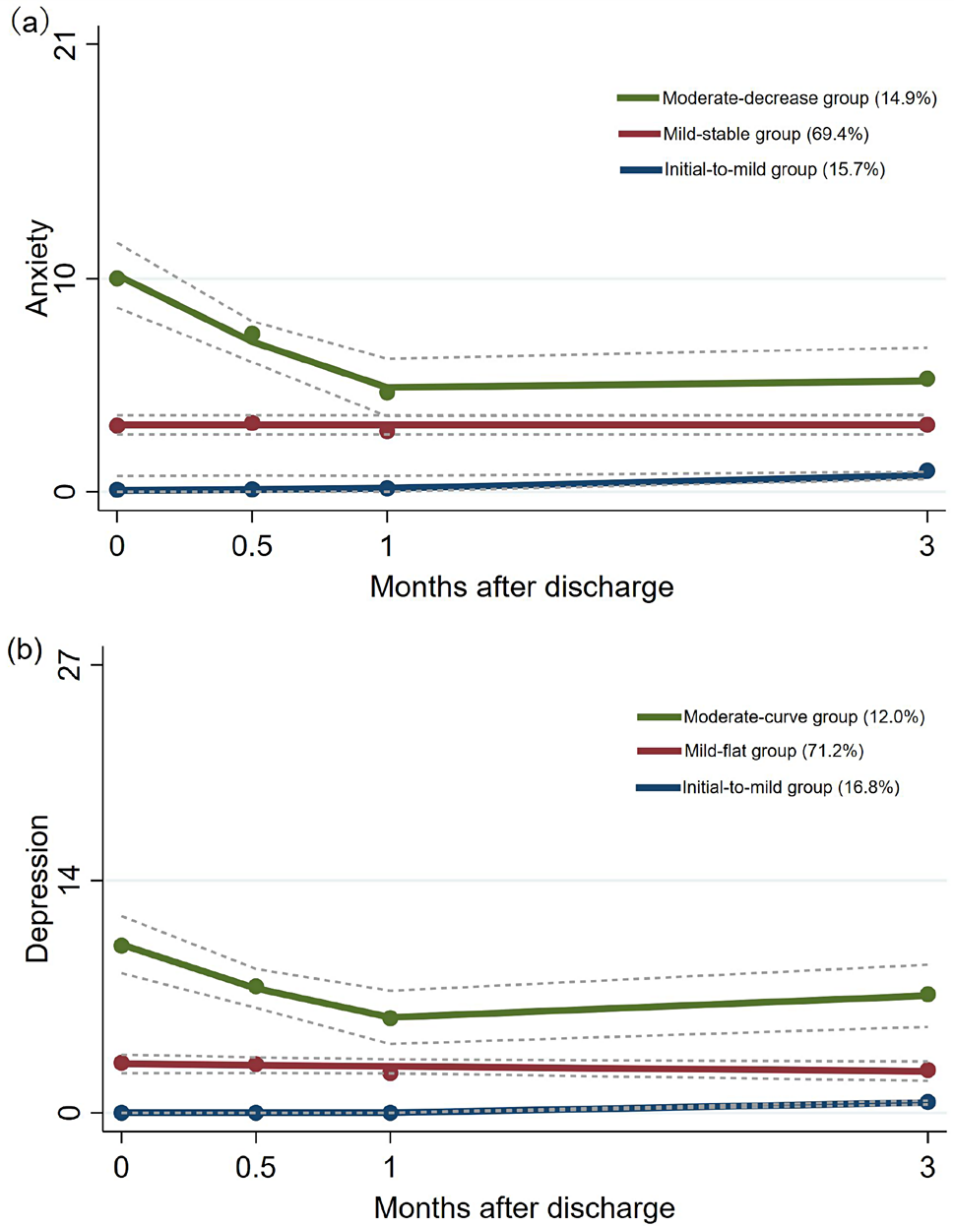
Caregiver anxiety and depression Trajectories (N = 267). (**a**) The caregiver anxiety trajectories and (**b**) depression trajectories. Solid lines represented the average estimated self-efficacy over time. Dashed lines represented the 95% confidence interval

ETable 2 shows the fit indices for the 1- to 5- class GBTM models of caregiver anxiety. The 3-class trajectory solution had the lowest information criteria values and an entropy value of 0.826. Based on the 3-class anxiety trajectory solution, 15.7% of caregivers were in the initial-to-mild group, 69.4% were in the mild-stable group, and 14.9% were in the moderate-decrease group (Fig. 3a). ETable 2 also shows the fit indices for the caregiver depression GBTM models. Based on the information criteria, entropy and interpretability, the 3-class model was selected as the best solution for depression trajectories. These were defined as initial-to-mild (16.8%), mild-flat (71.2%), and moderate-curve group (12.0%) (Fig. 3b). The anxiety and depression trajectory names were based on the GAD-7 and PHQ-9 questionnaire manuals for a clearer understanding.

## Dual trajectory models for caregiver self-efficacy with anxiety and depression

To examine the contemporaneous development of caregiver self-efficacy with anxiety and depression, we selected dual trajectory models with three latent trajectory groups of caregiver self-efficacy, anxiety and depression over the follow-up period.

**Fig. 4:**
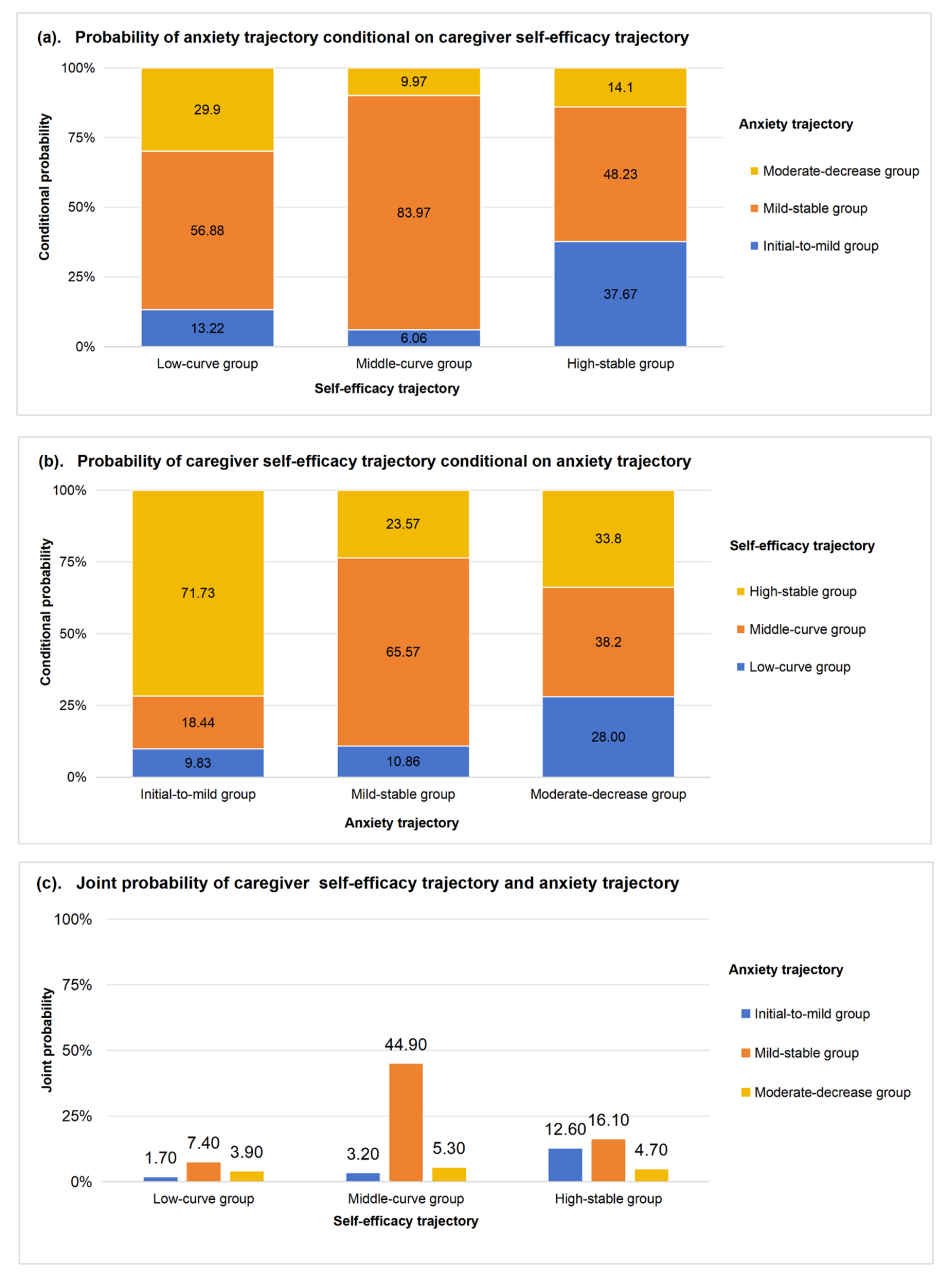
The linkage between caregiver self-efficacy and anxiety trajectory groups presented by conditional and joint probabilities from the group-based dual trajectory model

The conditional probabilities linking the caregiver self-efficacy and anxiety trajectories were shown in Fig. 4. Based on the conditional probability of anxiety given self-efficacy (Fig. 4a), the probability of following the moderate-decrease anxiety trajectory was the highest (29.9%) for those in the low-curve self-efficacy trajectory, compared to the other two self-efficacy trajectories. Additionally, caregivers in the high-stable self-efficacy trajectory were more likely to experience initial-to-mild anxiety compared to the other self-efficacy trajectories. Based on the conditional probability of self-efficacy given anxiety (Fig. 4b), 71.73% of those in the initial-to-mild anxiety group were also in the high-stable self-efficacy trajectory. Caregivers in the moderate-decrease anxiety group had similar probabilities of the three self-efficacy trajectories. Considering the 9 combinations of the anxiety and self-efficacy trajectories (Fig. 4c), only 12.6% of caregivers experienced the high-stable self-efficacy and initial-to-mild anxiety trajectories, while 44.9% caregivers experienced the middle-curve self-efficacy and mild-stable anxiety trajectories.

**Fig. 5:**
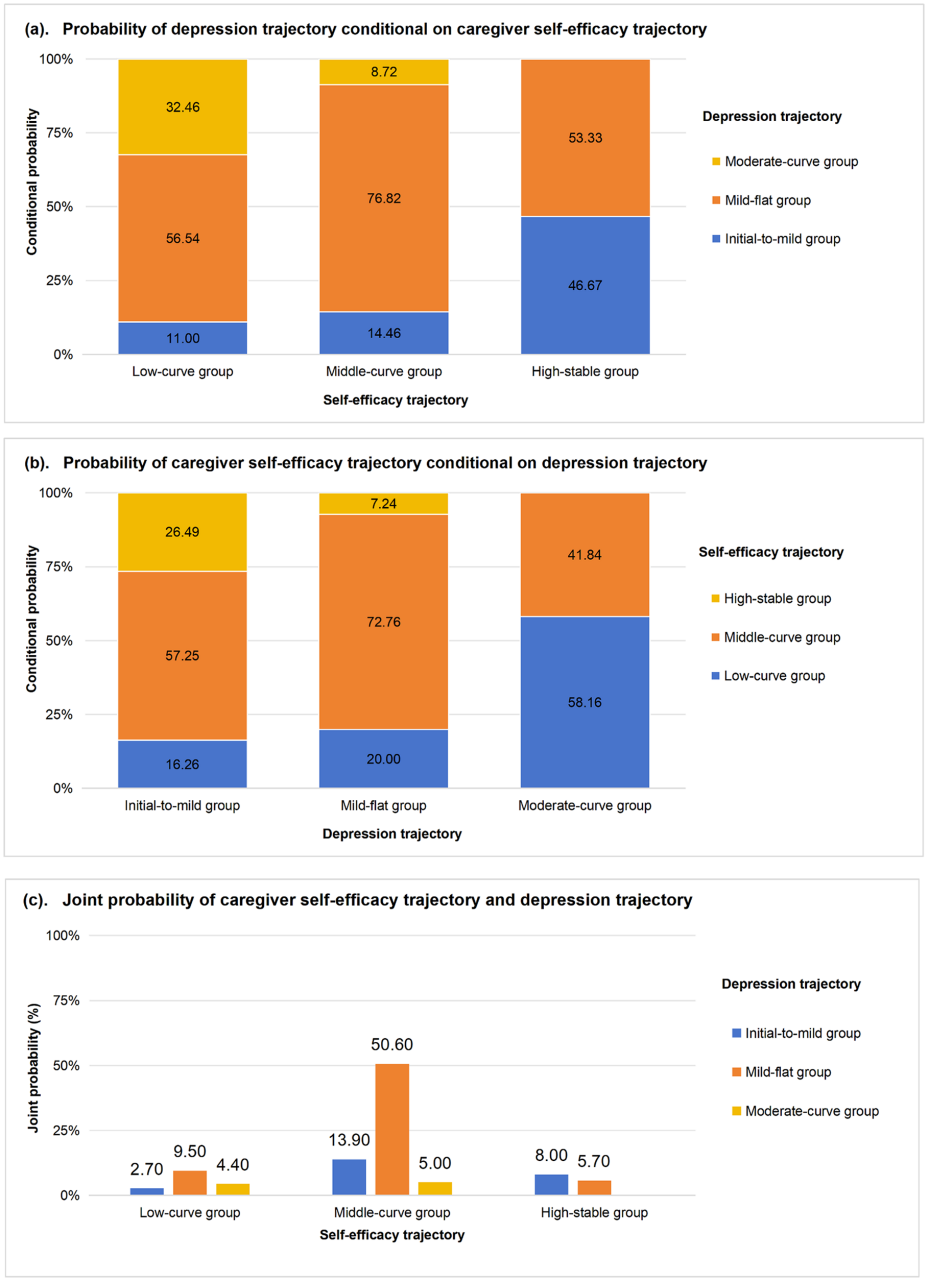
The linkage between caregiver self-efficacy and depression trajectory groups presented by conditional and joint probabilities from the group-based dual trajectory model

Figure 5 shows the temporal association between self-efficacy and depression. Figure 5a presents the probability of a depression trajectory conditional on caregiver self-efficacy trajectory. Caregivers in the initial-to-mild depression trajectory were more likely to belong to the high-stable self-efficacy trajectory compared to the other two self-efficacy trajectories. There was no probability of caregivers being in the moderate-curve depression trajectory if they had the high-stable self-efficacy trajectory. Regardless of self-efficacy trajectory, caregivers were most likely to experience the mild-to-moderate depression trajectory. Figure 5b shows that the low-curve self-efficacy trajectory still occurred in 16.26% of those with the initial-to-mild depression trajectory. Caregivers in the moderate-curve depression group had a zero probability of being in the high-stable self-efficacy trajectory. Approximately half of caregivers experienced the middle-curve self-efficacy and mild-flat depression trajectories, while only 8% of caregivers had the optimal combination of an initial-to-mild depression trajectory and a high-stable self-efficacy trajectory (Fig. 5c).

## Discussion

To our limited knowledge, this is the first study to explore the latent trajectories of self-efficacy and their associations with depression and anxiety trajectories in caregivers of patients with HF. Previous studies have established the association between caregiver self-efficacy and mental well-being, but the longitudinal co-development of these factors has been understudied.

Consistent with previous research that identified heterogeneous progression trajectories in adults [35], three distinct self-efficacy trajectories were observed in caregivers of patients with HF. In this study, approximately 1 in 14 caregivers exhibited a high-stable trend of self-efficacy during the three months after the patient’ s discharge, while the remaining caregivers showed varying lower degrees of self-efficacy. Strategies to improve caregiver self-efficacy should be an integral component of caregiver interventions [18]. About two-thirds of caregivers experienced a middle-curve self-efficacy trajectory. During the first month following the discharge of their patients, they exhibited a remarkable increase in confidence in assisting the patients with self-care. However, this confidence showed a gradual decline in the subsequent two months, ultimately converging to a level comparable to that observed at the time before discharge. In every four individuals, one caregiver belonged to the low-curve self-efficacy group, which, although showing a slight increase in the short-term post-discharge, still failed to surpass half of the total score on the CSE-CSC scale and remained lower than the average level reported in other countries [6]. Worse still, one month after discharge, these caregivers continued to face the risk of further declines in self-efficacy. Our findings suggested that interventions aimed at improving caregiver self-efficacy should prioritize the 1–3 months period after discharge of HF patients. Increasing the frequency and content of the interventions during this term may help ensure that the caregiver self-efficacy continues to improve after the first month post-discharge. Maintaining the positive trajectory established in the initial post-discharge period would be an ideal outcome for such caregiver-focused interventions. Furthermore, the intervention should allocate additional resources to the “disadvantaged” caregivers - those in the low-curve group. Providing extra support and interventions targeted at this more vulnerable subset could help ensure they experience improvements in self-efficacy. Therefore, our result may provide clinical evidence to guide the determination of intervention dosage and frequency for the future study, thereby informing evidence-based practice.

Several caregiver characteristics may help distinguish among the three self-efficacy trajectories: chronic conditions, work status and regular exercise. The low-curve group had a higher proportion of caregivers with chronic illnesses compared to the other two groups in our study. Similar to our study, Irene and colleagues found that caregiver physical health significantly predicted low self-efficacy in pain management for patients with cancer [36]. While our study did not prospectively test Bandura’s self-efficacy theory, the observed link between caregivers’physical health and self-efficacy resonates with the theory’s emphasis on physiological states as a potential modulator of efficacy judgments [37]. In our study, we observed a higher proportion of unemployment within the low-curve caregiver self-efficacy group compared to the other two groups. This finding aligns with a previous study in patients with coronary artery diseases, which highlighted the significance of occupation as an independent predictor of individual cardiac self-efficacy [8]. However, there was no difference in self-efficacy among the different occupational status caregivers in a cross-sectional study in Singapore [38]. The relationship between employment status and self-efficacy warrants further exploration, as it may have implications for interventions aimed at improving well-being of caregivers and the patients they care for.

Individuals who persisted in regular physical activity tend to manifest heightened self-efficacy. In our study, the majority of caregivers in the high-stable self-efficacy group adhered to regular exercise, but only less than a third in low-curve self-efficacy did. Furthermore, a previous study indicated that individuals with initially high levels of physical activity, even if declining, exhibited greater self-efficacy than those with low physical activity levels that were also declining [39]. However, when promoting physical exercise among caregivers, the issue of “time constraints” must be thoroughly considered. Caregivers often face significant challenges in maintaining their own health due to the heavy demands of their caregiving responsibilities. This phenomenon results in limited availability of time for self-care activities, including exercise. Future research is needed to create effective caregiver support regimes, with an emphasis on enhancing resources such as social support for caregivers and intergenerational family support [40, 41].

Regarding the relationship between the self-efficacy and mental health considering the temporal factor, previous study identified that lower self-efficacy skills made caregivers vulnerable for higher psychological distress in a long-term follow-up [42]. Our study provided a deeper insight into the relationship between caregiver self-efficacy and mental health based on their synergistic developmental pattern. In caregivers of patients with HF who had low-curve self-efficacy trajectory in our study, one in three experienced moderate level of depression or anxiety when the patients at discharge. Despite these caregivers continuing to experience relatively severe psychological distress in the 3-months after discharge, a surprising finding was that their anxiety and depression levels rapidly declined within the first month after discharge, without any additional resources or support provided beyond routine management. Caregivers had limited and inconsistent abilities to regulate the effects of anxiety and depression on their self-efficacy. In our study, despite one-third of the caregivers belonged to the most severe anxiety trajectory group, they were still able to attain the more ideal self-efficacy levels. But this phenomenon was not observed in the depression trajectory groups. Furthermore, caregivers with the initial-to-mild anxiety trajectory were most likely to exhibit high-stable self-efficacy, whereas those with the initial-to-mild depression trajectory had only a small probability of demonstrating the high-stable self-efficacy. This discrepancy in mental-regulatory abilities between anxiety and depression highlights the complexities involved in bolstering caregiver self-efficacy. Health promoters should not only delve into the trajectory profiles and population characteristics of caregiver self-efficacy, but also separately understand the difficulties experienced by caregivers of patients with HF with different trajectories of depression and anxiety. Furthermore, the ideal self-efficacy-mental health combinations were rare, meaning that there is much room for improvement in the direction in improving caregiver self-efficacy and their mental state. The results of our study suggested that future trials on caregiver self-efficacy should consider a priori whether the intervention should be targeted at a particular anxiety or depression trajectory group. Such an approach has the potential to facilitate personalized interventions and alleviate the “time constraints” faced by part of caregivers.

## Limitations

Several limitations of this study should be acknowledged. First, although we utilized a multi-center design to minimize sampling bias, the generalizability of the findings may be limited. When interpreting the results and their implications for nursing practice and caregiver health promotion, it is important to consider the unique multilingual clinical environments (e.g., dialect-speaking populations) as well as cultural context of the region. Second, our study aimed to elucidate the time-varying associations between caregiver self-efficacy, anxiety, and depression trajectories. We were unable to consider the underlying mechanisms of the interactions between self-efficacy, anxiety, and depression across different trajectory groups in this study, which will prompt further research. Third, despite including an adequate sample size, our study still faced missing data as a result of loss to follow-up or patient death. Although maximum likelihood estimation was used to handle missing data in conducting GBTM and GBDTM, it does not eliminate the possibility of bias. Lastly, we selected only four fixed-time points and relied on patient self-reported data to complete the assessment during the vulnerable period of patients with HF. Considering the meaningful results of this study, future qualitative or mixed method research is needed to reveal the state and characteristics of caregiver self-efficacy during HF patients’ vulnerable period. This may provide stronger evidence for improving caregiver self-efficacy and mental health.

## Conclusions

In conclusion, this study demonstrated three distinct trajectories of self-efficacy, anxiety, and depression among family caregivers of patients with HF. The dual-trajectory models revealed the probability of interrelationships between caregiver self-efficacy trajectories and those of anxiety and depression. The findings of this study have significant practical implications, providing an evidence base for healthcare providers to develop systemic strategies to enhance caregiver self-efficacy and mental health.

## Electronic supplementary material

Below is the link to the electronic supplementary material.

Supplementary Material 1

## Data Availability

The datasets used and/or analyzed during the current study are available from the corresponding author on reasonable request.

## Abbreviations

HF: Heart failure
STROBE: The Strengthening the Reporting of Observational Studies in Epidemiology
NYHA: New York Heart Association
GBDTM: Group-based dual trajectory modeling
GBTM: Group-based trajectory modeling
BIC: Bayesian information criteria
AIC: The Akaike Information Criteria
ANOVA: Analysis of variance
