# Supplementary material for: Co-occurring self-efficacy, anxiety, and depression in caregivers of patients with heart failure: A Group-Based Dual-Trajectory Modeling Approach
Source: BMC Nurs. 2025 Mar 31;24:351. doi: 10.1186/s12912-025-02995-0 (PMC11959719; doi:10.1186/s12912-025-02995-0)
Supplement: Supplementary file 1 — Supplementary Material 1 [file 12912_2025_2995_MOESM1_ESM.docx]

**ETable 1 Comparison of the main baseline characteristics for those included and excluded in the cohort**

| Characteristics | Included (N=267) | Excluded (N=32) | Characteristics | Included (N=267) | Excluded (N=32) |
| --- | --- | --- | --- | --- | --- |
|  | n (%)/ mean±SD | n (%)/ mean±SD |  | n (%)/ mean±SD | n (%)/ mean±SD |
| Caregivers |  |  | Patients they cared for | | |
| Age (years) | 58.3±13.1 | 58.2±12.9 | Age (years)* | 69.5±12.0 | 75.38±14.5 |
| Gender |  |  | Gender |  |  |
| Men | 103 (38.6) | 12 (37.5) | Men | 160 (59.9) | 22 (68.8) |
| Women | 164 (61.4) | 20 (62.5) | Women | 107 (40.1) | 10 (31.3) |
| Educational level* |  |  | Educational level |  |  |
| Elementary | 32 (12.0) | 14 (43.8) | Elementary | 59 (22.1) | 12 (37.5) |
| Middle school | 104 (39.0) | 17 (53.1) | Middle school | 128 (47.9) | 13 (40.6) |
| High school and above | 131 (49.1) | 1 (3.1) | High school and above | 80 (30.0) | 7 (21.9) |
| Life style^1^ |  |  | Medical insurance |  |  |
| Alcohol use | 46 (17.2) | 8 (25.0) | Having | 220 (82.4) | 27 (84.4) |
| Tobacco use* | 65 (24.3) | 6 (18.8) | No | 47 (17.6) | 5(15.6) |
| Regular exercise | 95 (35.6) | 10 (31.3) | NYHA class |  |  |
| Relationship to patients* | |  | Ⅰ or Ⅱ | 111 (41.6) | 9 (28.1) |
| Spouse/partner | 133 (49.8) | 8 (25.0) | Ⅲ | 116 (43.4) | 13 (40.6) |
| Child | 122 (45.7) | 20 (62.5) | Ⅳ | 40 (15.0) | 10 (31.3) |
| Other | 12 (4.5) | 4 (12.5) | Comorbidity counts |  |  |
| Times providing care to patient (years) * | |  | 1 | 45 (16.9) | 8 (25.0) |
| ＜3 | 34 (12.7) | 10 (31.3) | 2 | 75 (28.1) | 13 (40.6) |
| 3-6 | 95 (35.6) | 11 (34.4) | 3 | 73 (27.3) | 9 (28.1) |
| ＞6 | 138 (51.7) | 11 (34.4) | ≥4 | 74 (27.7) | 2 (6.3) |
| Work status^2^ |  |  |  |  |  |
| Manual work | 95 (35.6) | 12 (37.5) |  |  |  |
| Brain work | 49 (18.4) | 6 (18.8) |  |  |  |
| Retirement | 79 (29.6) | 8 (25.0) |  |  |  |
| No work | 44 (16.5) | 6 (18.8) |  |  |  |
| Residence* |  |  |  |  |  |
| Urban | 220 (82.4) | 22 (68.8) |  |  |  |
| Town/countryside | 47 (17.6) | 10 (31.3) |  |  |  |
| The presence of chronic diseases* | |  |  |  |  |
| No | 226 (84.6) | 21 (65.5) |  |  |  |
| Yes | 41 (15.4) | 11 (34.4) |  |  |  |

Abbreviations: NYHA class, New York Heart Association (NYHA) functional class.

^1^ Alcohol use was defined as drinking more than once a week, with each drinking session involving ≥50 ml of alcohol, and a drinking duration of more than 6 months. Tobacco used was defined as smoking ≥1 cigarette per day for a duration of more than 6 months. Regular exercise was defined as participating in aerobic exercise ≥3 times per week, with each session lasting ≥30 minutes.

^2^ Manual work included tasks primarily requiring physical exertion, such as agricultural production, craftsmanship, and workers in the service industry. Brain work included professional and technical work such as administrative personnel, salespeople, and office staff.

* represented a significant chi-square or t test analysis.

**ETable 2** Model fit statistics of the group-based trajectory modeling for 1 - to 5-class solutions (N=267)

| Class | Subgroup order | BIC | AIC | Entropy | Predicted probability of group membership |
| --- | --- | --- | --- | --- | --- |
| Caregiver self-efficacy | |  |  |  |  |
| 1 | 2 | -4218.50 | -4211.32 | NA | 1.00 |
| 2 | 2 2 | -4135.13 | -4120.78 | 0.705 | 36.00/64.00 |
| **3** | **2 2 0** | **-4118.73** | **-4100.80** | **0.782** | **25.09/67.79/7.12** |
| 4 | 0 2 2 0 | -4102.30 | -4080.78 | 0.811 | 1.65/33.52/58.97/5.86 |
| 5 | 0 2 2 1 0 | -4105.65 | -4078.75 | 0.799 | 1.62/31.63/55.34/4.79/6.62 |
| Anxiety |  |  |  |  |  |
| 1 | 2 | -2369.33 | -2362.136 | NA | 1.00 |
| 2 | 1 2 | -2303.00 | -2290.44 | 0.860 | 19.27/80.73 |
| **3** | **1 0 2** | **-2269.10** | **-2252.96** | **0.826** | **15.68/69.41/14.91** |
| 4 | 1 0 2 2 | -2271.39 | -2248.08 | 0.822 | 15.39/65.83/13.15/5.63 |
| 5 | 0 0 0 2 2 | -2278.12 | -2253.01 | 0.703 | 14.36/7.34/59.33/13.34/5.63 |
| Depression |  |  |  |  |  |
| 1 | 2 | -2268.71 | -2261.53 | NA | 1.00 |
| 2 | 0 2 | -2202.80 | -2192.04 | 0.603 | 56.66/43.34 |
| **3** | **1 1 2** | **-2165.03** | **-2147.09** | **0.916** | **16.82/71.19/11.99** |
| 4 | 1 0 2 1 | -2156.10 | -2134.58 | 0.917 | 16.62/67.45/15.56/0.37 |
| 5 | 1 2 1 2 1 | -2162.47 | -2131.98 | 0.872 | 16.59/60.44/19.12/3.48/0.37 |

*The model marked in bold was the optimum model for the trajectories of HF symptoms.
